# Chromosome-level genome assembly of *Tritrichomonas foetus*, the causative agent of Bovine Trichomonosis

**DOI:** 10.1038/s41597-024-03818-8

**Published:** 2024-09-20

**Authors:** Mostafa Y. Abdel-Glil, Johannes Solle, Daniel Wibberg, Heinrich Neubauer, Lisa D. Sprague

**Affiliations:** 1 Friedrich-Loeffler-Institut, Institut für Bakterielle Infektionen und Zoonosen (IBIZ), Naumburger Str. 96a, 07743 Jena, Germany; 2 Jena University Hospital – Friedrich Schiller University, Institute for Infectious Diseases and Infection Control, Jena, Germany; 3 Center for Biotechnology - CeBiTec, Bielefeld University, Universitätsstraße 27, D-33615 Bielefeld, Germany; 4 ELIXIR DE Administration Office, Institute of Bio- and Geosciences IBG-5, Forschungszentrum Jülich GmbH – Branch office Bielefeld, Universitätsstraße 27, D-33615 Bielefeld, Germany

**Keywords:** Eukaryote, Sequence annotation

## Abstract

*Tritrichomonas foetus* is a parasitic protist responsible for bovine trichomonosis, a reproductive disease associated with significant economic burden to the livestock industry throughout the world. Here, we present a chromosome-level reference genome of *T. foetus* -KV-1 (ATCC 30924) using short-read (Illumina Miseq), long-read (Oxford Nanopore) and chromatin-linked (Hi-C) sequencing. This is the first chromosome-level genome of a parasitic protist of the order Tritrichomonadida and the second within the Parabasalia lineage, after *Trichomonas vaginalis*, the human-associated causative agent of the sexually transmitted infection in humans. Our constructed genome is 148 Mb in size, with a N50 length of the scaffolds of 22.9 Mb. The contigs are anchored in five super-scaffolds, corresponding to the expected five chromosomes of the species and covering 78% of the genome assembly. We predict 41,341 protein-coding genes, of which 95.10% have been functionally annotated. This high-quality genome assembly serves as a valuable reference genome for *T. foetus* to support future studies in functional genomics, genetic conservation and taxonomy.

## Background & Summary

Bovine trichomonosis is a worldwide occurring, to the WOAH/OIE notifiable, venereal disease of cattle. The causative agent is the flagellate-like parasite *Tritrichomonas (T.) foetus*^1^. This parasite exists exclusively in the trophozoite stage and reproduces by binary longitudinal fission, without sexual reproduction. It colonises the epithelial surface of the lumen and crypts of the prepuce and penis and is transmitted during mating by asymptomatic bulls^2^. Infected cows and heifers, in contrast, present with premature embryonic death, uterine discharge, pyometra, irregular oestrus cycles and infertility^3,4^. The resulting reproductive failure not only drastically reduces the breeding efficiency in dairy and beef cattle^2^, it is also accompanied by significant financial damage due to reduced milk and calf production. According to estimates the losses caused by *T. foetus* may amount to around 1 billion US dollars per year in the US alone^5,6^.

*T. foetus* has been observed in other animal species such as domestic cats, horses and roe deer as well as swine; goats, dogs, rabbits and guinea pigs can be experimentally infected^7^. Human *T. foetus* infections causing meningoencephalitis and peritonitis in immunocompromised and immunosuppressed individuals have also been reported^8^. To date, the taxonomy of trichomonad parasites including the avian pathogen *Trichomonas (T.) gallinae*, the human parasitic pathogens *Pentatrichomonas hominis*, *T. tenax* and *T. vaginalis* has not been extensively studied. It is still a matter of debate to what is a true species as observed for *T. suis*, a commensal of pigs, and bovine *T. foetus*. Both parasites are indistinguishable morphologically, serologically and antigenically^7^. No chromosome-level genome for *T. foetus* exists so far and the two publicly available genome assemblies of *T. foetus* are too highly fragmented to obtain a descriptive reference genome needed for genetic studies, genomics and taxonomy^9,10^.

The present study describes the application of Illumina short-read, nanopore long-read and Hi-C sequencing methods to construct the genome of *T. foetus* KV-1 (ATCC 30924) at the chromosome-level. The *T. foetus* KV-1 genome is 148 Mb-long with an N50 scaffold length of 22.9 Mb, with 115 Mb of the assembled genome sequences mapped to five chromosomes. A total of 41,341 protein-coding genes were discovered. We incorporated the analysis steps described below into a fully automated pipeline for the characterisation of *T. foetus* genomics. This open-source pipeline is available at https://gitlab.com/FLI_Bioinfo/tricho-workflow and should enable laboratories not only to replicate our analytical approach, but also to apply this approach to other *T. foetus* strains. Although we present a high-quality genome at the chromosome scale, we anticipate that ongoing advancements in sequencing technologies, as well as assembly and annotation algorithms, will continue to enhance the precision and completeness of this genomic dataset.

## Methods

### *T. foetus* KV-1 sequencing and data pre-processing

Genomic DNA was obtained from *T. foetus* KV-1 cultivated in InPouch® TF-Bovine (Megacor; Austria) to a concentration of 1.5 *10^6^/mL using the UNSET lysis buffer and subsequent phenol: chloroform extraction method as described in^11^. The DNA quality was assessed using a NanoDrop Spectrophotometer (Thermo Fisher, USA), and quantified by a Qubit Fluorometer (Invitrogen, USA). For long-read sequencing, we used the R9.4.1 sequencing chemistry from Oxford Nanopore Technologies (ONT) on a GridIon platform with the ligation kit SQK-LSK109. The ONT data were collected and base-called with Guppy 6.2.11 using the sup-accurate model. This resulted in a cumulative data set of 1.3 million reads with a total length of 9.40 billion bases and an average length of 7.05 kb. Subsequent trimming and filtering of the ONT data using Porechop^12^ (default mode) and filtlong^13^ (-min_length 1000), respectively, yielded 826,311 reads with an average length of 11 kb and cumulative base count of 9 billion bases (Table 1). Illumina sequencing was done on a MiSeq machine (Illumina, USA) using the XT DNA Library kit, generating paired-end short reads of 300 bp. After Illumina sequencing, 52 million reads with a cumulative base count of 12.3 billion bases of raw sequences were obtained for *T. foetus* KV-1. The accuracy of the Illumina reads was improved by performing adapter trimming and quality control with fastp (v0.23.2)^14^. This left a total of 51.4 million reads with a total number of 11.9 billion bases and a Q30 of 88.3%.

**Table 1:** Statistics of raw and quality filtered sequencing data used for the assembly.

Sequenicng libraries (Platform)	Number of reads	Number of bases	Average read length	Maximum read length	Q30(%)
WGS long reads (ONT GridION)	1.333.158	9.401.571.876	7052,1	253421	30,67
WGS long reads + Quality filtering*	826.311	9.093.615.268	11005,1	253392	30,79
WGS short reads (Illumina MiSeq)	52.517.758	12.309.658.911	234,4	301	86,63
WGS short reads + Quality filtering*	51.428.046	11.900.989.860	231,4	301	88,29
Hi-C (Illumina MiSeq)	49.625.472	14.937.267.072	301	301	81,02
Hi-C + Quality filtering*	45.730.418	12.270.344.892	268,3	301	89,64

*Quality filtering of the nanopore reads employed Porechop (default settings) and filtlong (min_length = 1000 bp) while quality filtering for the Illumina genomic DNA and Hi-C libraries employed fastp program (default parameters).

### *De novo* genome assembly, Hi-C scaffolding and quality assessment

The *de novo* genome assembly was based on long-read assembly followed by polishing with short reads to ensure the contiguity and accuracy of the genome. The long-read assembly was based on nanopore reads with a length of at least 1000 bases. We then evaluated the performance of the four different assemblers on our data: flye^15^ (v 2.9), wtdbg2^16^ (v2.5), Raven^17^ (v1.8.3) and Shasta^18^ (v0.11.1) (Table S1). The predicted genome size of the respective assemblers varied between 109 and 220 Mb, as did the contiguity of the genomes in terms of number of contigs (range 207 to 5992), contig N50 (range 68 Kb to 2.6 Mb) and maximum contig size (range 916 Kb to 10,7 Mb) (Table S1). The assembly results of the two assemblers Wtdbg2 (producing the largest genome with high fragmentation; Table S1) and flye (smallest genome with improved contiguity; Table S1) were merged using quickmerge^19^ (v0.3) based on a sequence overlap of >5 kb between assemblies. The flye assembly was then used as a query and the wtdbg2 assembly as a reference in order to create larger, contiguous genome segments. Multiple rounds of assembly polishing were subsequently performed, first with long-read using two iterations of racon^20^ (v1.5.0; with minimap2^21^ v2.24), followed by two rounds of short-read polishing with the fastp-driven Illumina reads using Polypolish^22^ (v0.50; with bwa v0.7.17). A total of 339,488 substitution errors in the initial assembly were corrected with Illumina data resulting in a final consensus quality of 99.9%. We estimated the average read depth per contig by mapping the nanopore reads to the polished contigs using minimap2^21^ (v2.24), followed by qualimap^23^ (v 2.2.2a) to evaluate the mapping quality. Finally, we discarded the contigs with a coverage depth of less than 7-fold. This approach resulted in a total number of 670 contigs (total size 148,7 Mb) which were then selected for scaffolding.

**Fig. 1:**
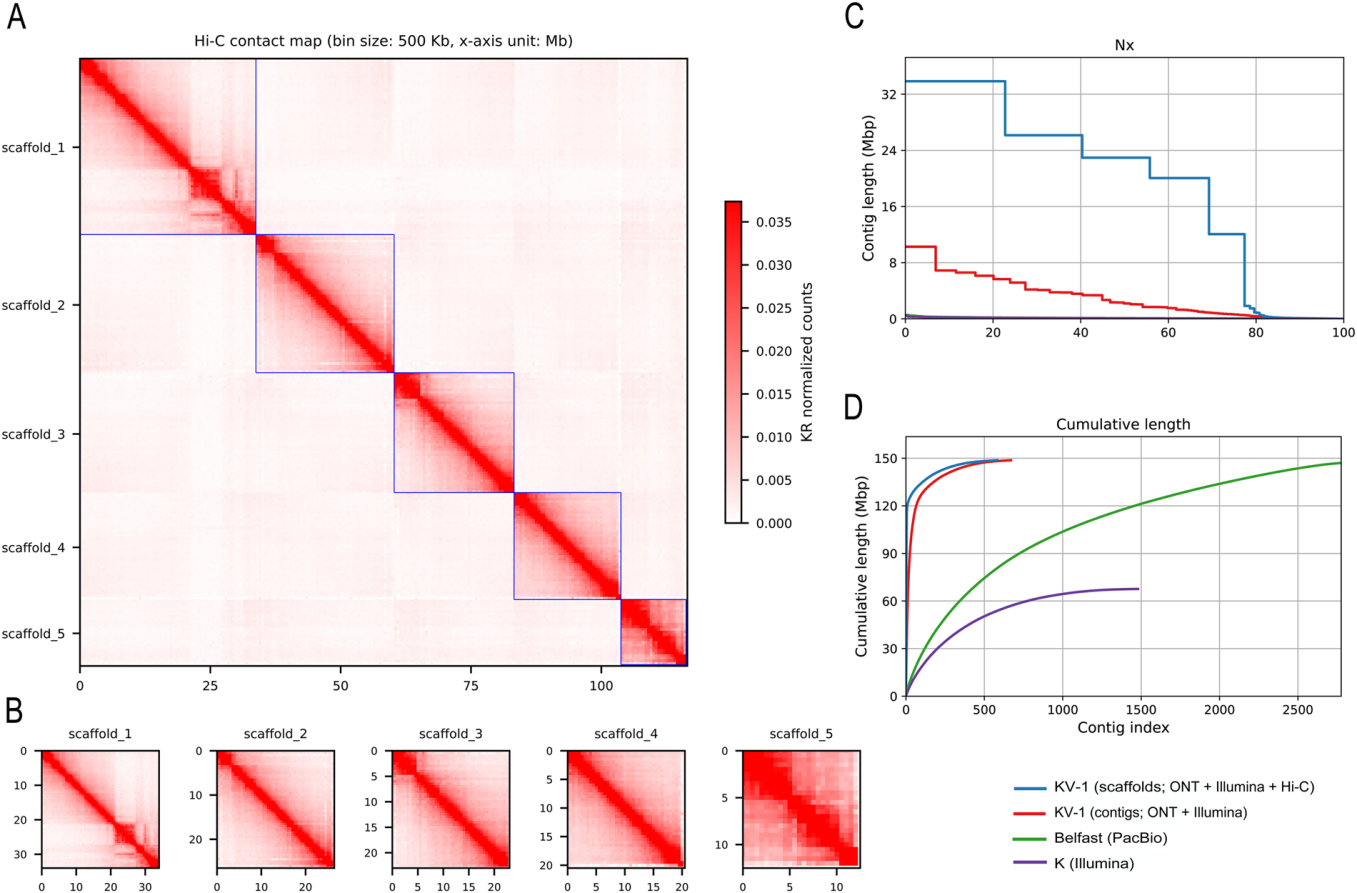
Results of Hi-C Scaffolding of *T. foetus* KV-1 genome assembly. (**A**) Genome-wide and (**B**) separate plots of the Hi-C interaction mapping of the five chromosome-level scaffolds in the *T. foetus* KV-1 Hi-C assembly. (**C,D**) Quality control results showing the contiguity parameters of the Hi-C scaffold and contigs of the *T. foetus* KV-1 genome generated in the study, compared to other available genomes of *T. foetus* at NCBI. The results indicate improved contiguity of our genome assembly.

To create a chromosome level assembly, Hi-C proximity ligation data were used to anchor, order and orient the assembled contigs. *In-situ* Hi-C sequencing was accomplished with the EpiTect Hi-C Kit (Qiagen). In brief, two *in-situ* Hi-C libraries were prepared, including steps for cell crosslinking, cell lysis, chromatin digestion, biotin labelling, proximal chromatin DNA ligation and DNA purification. Hi-C sequencing using the Illumina MiSeq machine generated a total of 49.6 million raw reads with a base count of 14.9 billion bases. After data filtration with fastp (v0.23.2), a total of 45.7 million clean reads with a base count of 12.3 billion bases were retained. The ARIMA Genomics Hi-C Mapping Pipeline (https://github.com/ArimaGenomics/mapping_pipeline; accessed February 2024) was then applied to identify pairs of reads originating from physically interacting genomic regions. This pipeline aligns Hi-C reads with bwa-mem^24^ (v0.7.17) in single-end read mode, followed by trimming the 3′ end of reads marked as chimeric or spanning ligation junctions. Paired reads were filtered based on the mapping quality using samtools^25^ (v1.19.2) and PCR duplicates were eliminated with Picard^26^ (v 3.5.3). As no reference was available, YAHS^27^ (Yet another Hi-C scaffolding tool, v1.2a.2) was used to assemble the draft genome into a chromosome candidate utilising aligned Hi-C data in BAM format. Visualisation of the assembler’s Hi-C maps was obtained using HapHic plot^28^ (v1.0.3) (Fig. 1).

**Fig. 2:**
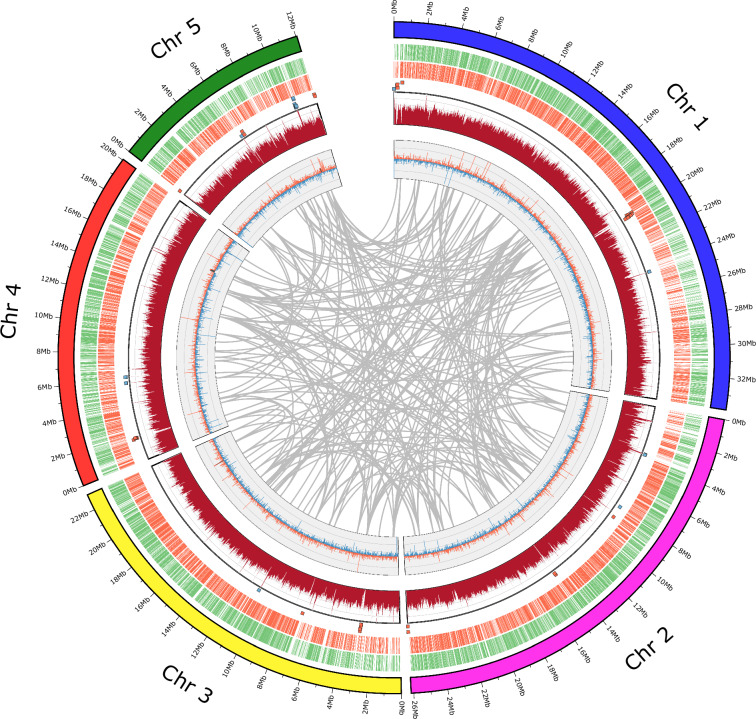
Circos plot depicting the genomic features of *T. foetus* KV-1. The five large scaffolds of the genome are shown on an MB scale (circle 1) followed by representation of the predicted annotated coding genes in clockwise (circle 2) and anticlockwise (circle 3) orientations. Circles 4 and 5 show the coverage pattern of short-reads and long reads across the genome, respectively. Genomic areas with abnormal coverage are highlighted with blue or red squares, denoting for excessively high (>150-fold) or low (<15-fold) coverage, respectively. Circle 5 shows repeat regions in-between the five scaffolds that are more than 20 kb in size.

The final genome assembly of *T. foetus* KV-1 comprised 148,7 Mb, with Hi-C analysis revealing five super-scaffolds (sizes 12, 20, 22, 26 and 33 Mb) corresponding to the expected five chromosomes of *T. foetus*^29,30^ (Fig. 1). The scaffolds anchored 78% (115 Mb) of the genome assembly, with a total N50 of 22.9 Mb and 2.18 Mb for scaffold and contig lengths, respectively. The average GC content of the genome assembly was 30.75% (Table 2, Fig. 2).

**Table 2:** Statistics of the genome assembly and gene structure annotation of *Tritrichomonas foetus* KV-1.

Elements	Metric	Value
**Genome Assembly**	Number (n.) of scaffolds (contigs)	5/670
	Total length of assembly (Mb)	148,7
	Total length of scaffolds (Mb)	115 Mb (78%)
	G + C content (%)	30,75%
	Average coverage (with long reads)	64,78-fold
	Average coverage (with short reads)	79,56-fold
	N50 scaffold/contig length (Mb)	22,9/2,18
	n. of gaps %	11200 (0.01%)
**Genome Scaffolds**	Scaffold 1	Length = 33.8 Mb; Cov. = 69X; GC% = 31.22%; Gaps = 1600
	Scaffold 2	Length = 26,2 Mb; Cov. = 70X; GC% = 30.73%; Gaps = 2400
	Scaffold 3	Length = 22,9 Mb; Cov. = 69X; GC% = 30.68%; Gaps = 1800
	Scaffold 4	Length = 20 Mb; Cov. = 69X; GC% = 30.77%; Gaps = 700
	Scaffold 5	Length = 12 Mb; Cov. = 74X; GC% = 30.76%; Gaps = 1600
**Annotation**	n. protein-coding genes	41341
	n. monoexonic genes	36167
	n. multiexonic genes	3035
	Average gene length (bp)	1490
	Average exon length (bp)	1349
	Average intron length (bp)	175
	Average exon number per gene	2
	n. tRNA genes	377
	n. 5.8S, 18S, and 28S rDNA units	133

**Fig. 3:**
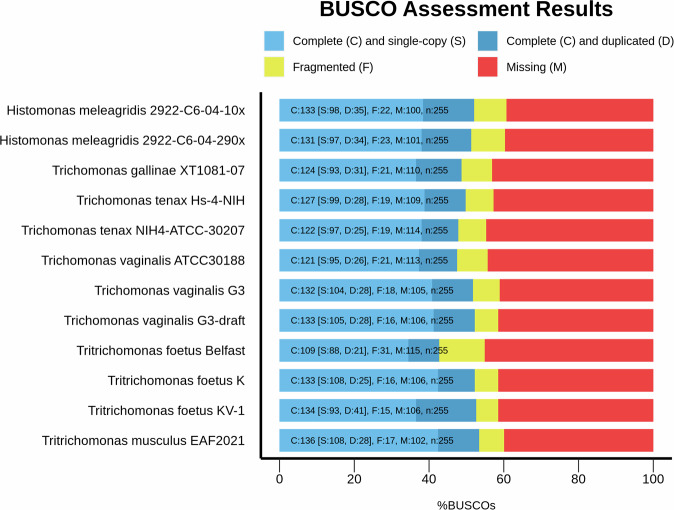
Comparison of the BUSCO assessment of genome completeness for the *T. foetus* KV-1 genome assembled in this study in comparison to all other publicly available genomes of Parabasalia using the eukaryote_odb10 data set.

This final version of the genome assembly of *T. foetus* KV-1 showed a considerable improvement over other publicly available *T. foetus* genomes based only on Illumina^9^ (strain K; accession, GCA_001839685.1^31^) or PacBio data^10^ (strain Belfast; accession, GCA_905133005.1^32^) (Table S2). Our assembly showed high contiguity with an improved scaffold N50 of 22 Mb. Table S2 shows genome statistics of the assembled genome along with the other publicly-available Parabasalia genomes at NCBI. The *T. foetus* KV-1 genome assembled in the present study is comparable in size with the *T. foetus* Belfast genome (146 Mb)^15^. The estimated genome size of *T. foetus* KV-1 is smaller than the genome size of *T. vaginalis* (160–180 Mb)^33^. We confirmed the smaller genome size for *T. foetus* KV-1 by flow cytometry (Supplementary Fig. S1). We also confirmed the absence of bacterial or archaeal contamination in our assembly (Supplementary Fig. S2). Finally, we compared our genome to all other genomes of the order Parabasalia using a set of 255 marker genes (Benchmarking Universal Single-Copy Orthologs; BUSCOs^34^ v5.5.0) from the eukaryota_odb10 dataset to assess the completeness results of our genome. A completeness score of 52.6% (138 complete BUSCOs) was obtained. This completeness value for *T. foetus* KV-1 is in a similar range as all the other Parabasalia genomes (Table S2; Fig. 3), suggesting that some integrated eukaryotic BUSCO markers may be missing in Parabasalia or that the gene prediction method applied by BUSCO is currently insufficient for these genomes. The recently described tool OMARK (v0.3.0) with the Eukaryota ancestral clade revealed a completeness of 66.3% (660 of the 995 conserved Hierarchical Orthologous Groups, HOGs). The analysis showed that 26.13% of these HOGs were single-copy, 40.2% were duplicated, and 33.67% were missing. Whole proteome analysis revealed that 14,343 out of 41,341 proteins (34.69%) showed consistent lineage placement.

#### K-mer-based approximation of genome size

The k-mer-based approximation (kmer length between 11 and 33-mers) of the genome size of *T. foetus* KV-1 using Illumina data resulted in a genome size of ~58 Mb. This approximation employed Jellyfish^35^ (v2.3.09) and GenomeScope^36^ (v1.0.08), with an estimated heterozygosity rate of ~1.86% (Supplementary Fig. S3). This underestimation of the genome size might be caused by the complexity of the genome, non-uniform coverage of the genome structure, or an excessive abundance of highly repetitive regions.

### Repeat annotation

Repetitive sequences were identified through a combination of *de novo* and structure-based predictions. We predicted tandem repeats with the Tandem Repeats Finder tool^37^ (v4.09) (Table 3). In addition, we used RepeatModeler2^38^ (v2.0.5) with the underlying tools, RECON (v1.08), RepeatScout (v 1.0.6), LTR_retriever (v2.9.0), and LTRharvest (genometools v1.6.4), to construct *de novo* species-specific transposable elements (TE) libraries for repeat annotation. This TE library was subsequently employed by RepeatMasker^39^ (v4.1.6; https://www.repeatmasker.org/) for the identification of both known and novel TEs in the *T. foetus* KV-1 genome. The *T. foetus* KV-1 genome was highly enriched for repetitive elements with a total of 60.84 Mb identified as repeat elements representing 48.93% of the full genome. These repeat elements were distributed unevenly along the scaffolded chromosomes. Of the repetitive elements, DNA transposons were the most common and accounted for ~30% of the genome. Other categories of repetitive sequences included retroelements (2.85%), LTR elements (2.28%), LINEs (0.56%) and simple repeats (0.95%). A high proportion (16.19%) of identified repeat elements were unclassified which may be accounted by the lack of studies on the repeats in Parabasalia. Additionally, minor proportions of repetitive elements were attributed to rolling-circles and SINEs. Finally, the total number and length of microsatellites predicted in *T. foetus* KV-1 with Krait^40^ (v1.5.1) were 69,977 and 5.393,631 bp, respectively.

**Table 3:** Repeat content analysis of the *T. foetus* KV-1 genome.

Repeat elements	Count	Total Length (bp)	Proportion in genome (%)
**Interspersed repeats**			
SINE	118	19701 bp	0,76
LINE	101	330525 bp	0,22
LTR	1714	777456 bp	0,52
DNA transposons	20695	48379606 bp	32,53
Rolling-circles (RC)	0	0 bp	0
Unclassified	33697	25079482 bp	16,86
Small RNA:		0 bp	0
Total interspersed repeats:		74586770 bp	50,15
**Tandem repeats**			
Tandem repeat finder #	69977	13609634 bp	9,15
Microsatellites (SSRs, iSSRs) *	165361	5393631 bp	3,62
Simple repeats:	23238	1214291 bp	0,82
Low complexity:	5875	354663 bp	0,24

#predicted with trf

*Microsatellites predicted with krait.

### Annotation of non-coding RNA genes

Infernal^41^ (v1.1.5) was used to search for homologues of structural RNAs through alignment with the Rfam RNA database^42^ (v14.9) (http://rfam.xfam.org/). In this release of the Rfam database, 4108 RNA families represented by covariance models (CMs) are available. Searching the Rfam CMs with cmscan^41^ (v1.1.5) identified 517 high-scoring structural RNAs hits in the *T. foetus* KV-1 genome including 377 transfer RNAs (tRNAs) and 133 ribosomal RNAs (rRNAs) (params:–cut_ga–nohmmonly; removing the hits with high-scoring-overlaps; Table 2, Table S3).

### Gene prediction

The *de novo* prediction of the protein-coding genes was done using BRAKER^43–53^ (v3.0.8) based on the repeats-soft-masked genome from RepeatMasker and the OrthoDB^54^ database of protein sequences. BRAKER applies GeneMark-ES (v4.72), with long genes predicted by GeneMark-ES serving as input for training AUGUSTUS (v3.5.0)^44^. Then, *ab initio* prediction of gene structures is performed with AUGUSTUS^44^. Table 3 shows the annotation features of the *T. foetus* KV-1 genome. In total, 41,341 protein-coding genes were identified, including 36,149 monoexonic and 3,035 multi-exonic genes. A maximum of two exons was identified per gene. The average gene length was 1490 bp (range 164 to 29,276 bp). The average exon length was 1349 bp (range 2 to 14942 bp). The average intron length was 175 bp (range 5–898 bp). Figure 2 shows the density of predicted genes, repetitive sequences, and alignment rates of the sequencing reads across the five chromosomes of the *T. foetus* KV-1 genome. Together, these results offer a foundation for further analysis of the genetic architecture of *T. foetus*.

### Functional genome annotation

**Fig. 4:**
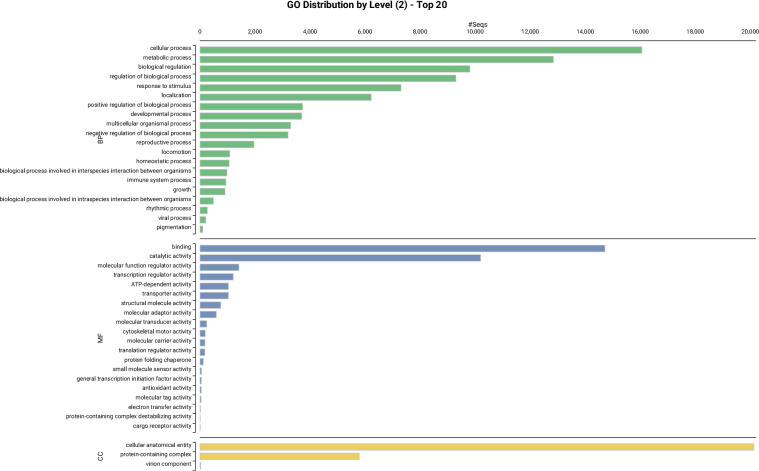
Bar plots depicting the Gene Ontology (GO) classification of functionally annotated genes in the Hi-C assembly. The distributions of GO terms are presented within the three main GO domains including “biological process” (green), “molecular function” (blue), and “cellular component” (yellow).

Functional genome annotation was performed by comparing the protein sequences of the predicted genes with the functional databases NR protein database, SWISS-PROT^55^, Gene Ontology (GO)^56^, eggNOG^57^ and KEGG^58^ using DIAMOND Blastp^52^ (e-value 1.0e-3) through the Functional Analysis Module of OmicsBox^59^ (v3.0.30). Additionally, InterProScan^60^ (v5.69–101.0) was used with the EMBL-EBI version of InterPro^61^ for the functional analysis of proteins (Table S4). Of the 41,341 predicted protein sequences, 37,751 had BLAST hits in the NR database, 31,954 in the RefSeq database and 19,555 in the SWISS-PROT database. 41,339 predicted protein sequences were identified with InterProScan. 24,714 (59.7%) predicted protein sequences were mapped with Gene Ontology Terms, of these, 23,494 (56.8%) sequences with Enzyme Code Annotations. 26,868 (53%) of the predicted sequences generated eggNOG results and could be assigned to OG functional Categories. The KEGG database identified 411 pathways, with 11,905 protein sequences linked to these pathways, while the reactome database^62^ identified 2480 pathways including 2223 sequences. The GO classification of the functionally annotated genes within three main GO domains showed that 49.5% (n = 21,066) of the genes belong to “biological process”, 51% (n = 21,680) to “molecular function”, and 61,9% (n = 26,318) to “cellular component” (Fig. 4). The most prominent GO terms at level 2 for biological process classes were “cellular process” (39%, n = 16,064) and “metabolic process” (31%, n = 12847). Similarly, for the cellular component domain, the main GO classes identified were “cellular anatomical entity” (49%, n = 20 134) and “protein-containing complex” (14%, n = 5786). For molecular function GO terms at level 2, “binding” (36%, n = 14,719) and “catalytic activity” (25%, n = 10,201) were the primary classes involved (Table S5).

### Data reproducibility through automated workflow

In addition to the *T. foetus* KV-1 strain sequenced in this study, it is to be expected that more high-quality genome sequences will become available for this species. The availability of multiple genome sets will considerably increase the knowledge on genetic diversity within *T. foetus* strains of different geographic origin and animal hosts. To ensure data reproducibility and facilitate future genome studies, we have developed an automated, open-source, Snakemake^63^ pipeline for the analysis of *T. foetus* genomes, available at https://gitlab.com/FLI_Bioinfo/tricho-workflow. This pipeline incorporates the assembly and annotation steps used in this work.

## Data Records

All raw data of the whole genome of *T. foetus* KV-1 have been deposited at the National Center for Biotechnology Information (NCBI) under the BioProject accession number PRJNA1123626^64^; BioSample accession number SAMN41816898. The short and long read sequences have been deposited in the SRA accessions SRR29430452 and SRR29430454 respectively. The Hi-C sequencing data have been deposited in the SRA accession SRR29430453. The final genome assembly has been deposited in GenBank at JBEJVY000000000.

## Technical Validation

To validate the genome assembly, QUAST^65^ (v5.0.2) was utilised to report assembly contiguity metrics, followed by BUSCO^34^ (v5.1.2), compleasm^66^ (v0.2.6) and OMArk^67^ (v0.3.0) to assess the completeness of the final scaffolded genomes. Additionally, we calculated the percentage of short reads mapping to the final scaffolded assembly. For this, Illumina data were trimmed with fastp^14^ and mapped using bwa mem^24^, and mapping quality was assessed with Qualimap^23^. Of the Illumina QC-passed reads, 99.98% (n = 24,967,332) were primarily mapped, with 99.65% (n = 24,831,090) properly paired. Similarly, long-read sequencing data were mapped to the genome, resulting in a mapping rate of 99.90%. Finally, the genome assembly with SPAdes^68^, that was based solely on Illumina reads, was fully contained within the final assembly from the long-read data. No evidence of contamination with foreign DNA from a different taxon was detected in the assembly based on metagenomics binning using MetaBAT2^69^.

## Supplementary information

Figure S2

Figure S3

Figure S1

Supplementary Tables

## Data Availability

All software and tools in this study were used with their default parameters, unless otherwise detailed. The workflow used for the assembly and annotation is available at https://gitlab.com/FLI_Bioinfo/tricho-workflow.
