# Supplementary material for: Chromosome-level genome assembly of Tritrichomonas foetus, the causative agent of Bovine Trichomonosis
Source: Sci Data. 2024 Sep 20;11:1030. doi: 10.1038/s41597-024-03818-8 (PMC11415386; doi:10.1038/s41597-024-03818-8)
Supplement: Supplementary file 1 — Figure S2 [file 41597_2024_3818_MOESM1_ESM.pdf]

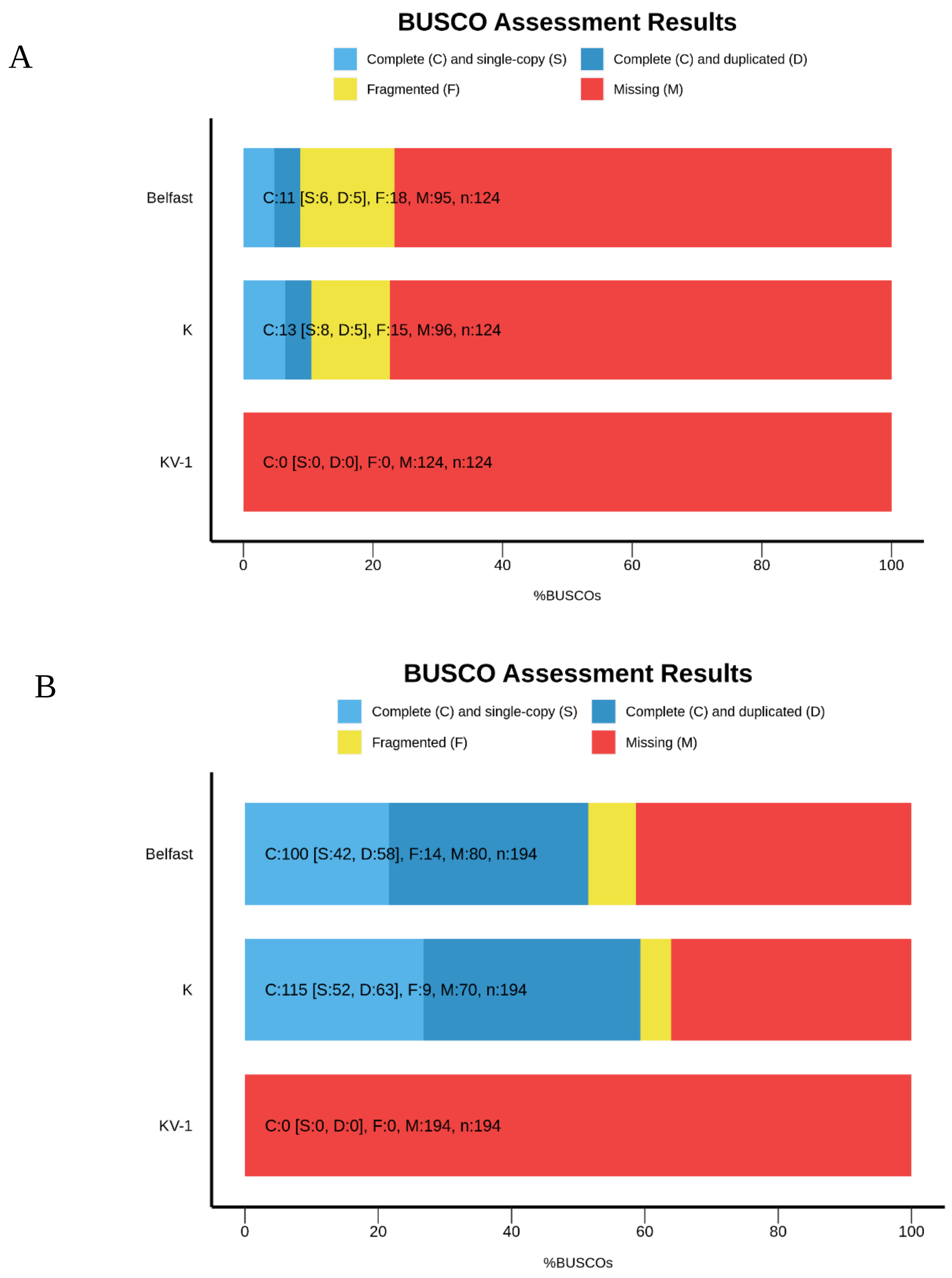

Figure S2: Comparison of the BUSCO assessment of genome completeness for the *T. foetus* KV-1 genome assembled in this study in comparison to *T. foetus* K and *T. foetus* Belfast strains using the bacteria_odb10 (A) and archaea_odb10 (B) data sets.
