# Supplementary material for: Chromosome-level genome assembly of Tritrichomonas foetus, the causative agent of Bovine Trichomonosis
Source: Sci Data. 2024 Sep 20;11:1030. doi: 10.1038/s41597-024-03818-8 (PMC11415386; doi:10.1038/s41597-024-03818-8)
Supplement: Supplementary file 2 — Figure S3 [file 41597_2024_3818_MOESM2_ESM.pdf]

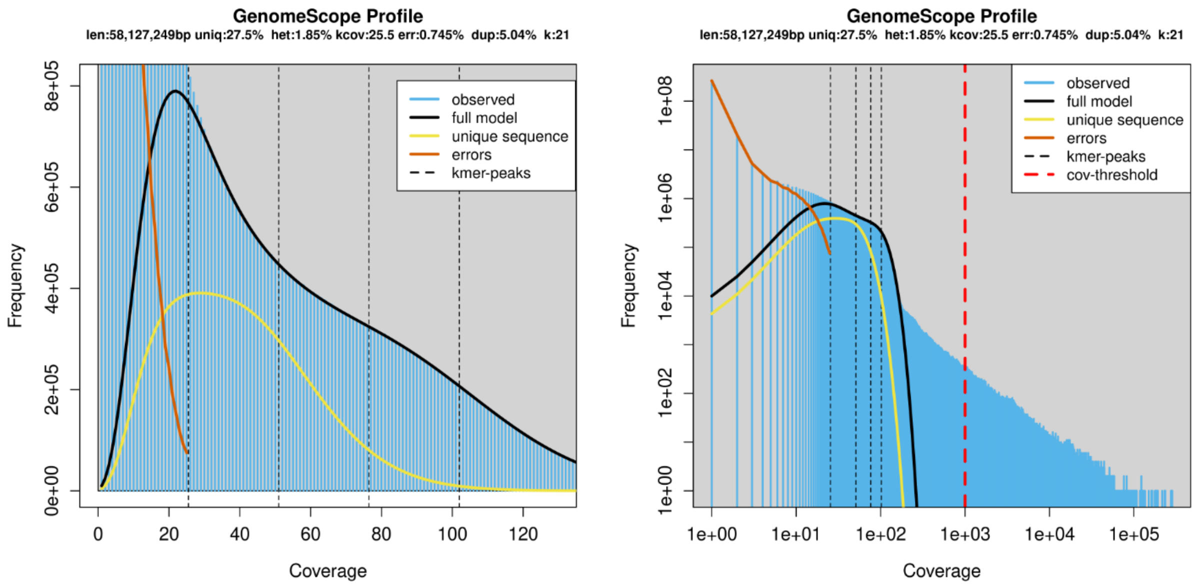

Figure S3: Estimation of the genome size of T. foetus KV-1 genome based on k-mer coverage distribution
