# Supplementary material for: Chromosome-level genome assembly of Tritrichomonas foetus, the causative agent of Bovine Trichomonosis
Source: Sci Data. 2024 Sep 20;11:1030. doi: 10.1038/s41597-024-03818-8 (PMC11415386; doi:10.1038/s41597-024-03818-8)
Supplement: Supplementary file 3 — Figure S1 [file 41597_2024_3818_MOESM3_ESM.pdf]

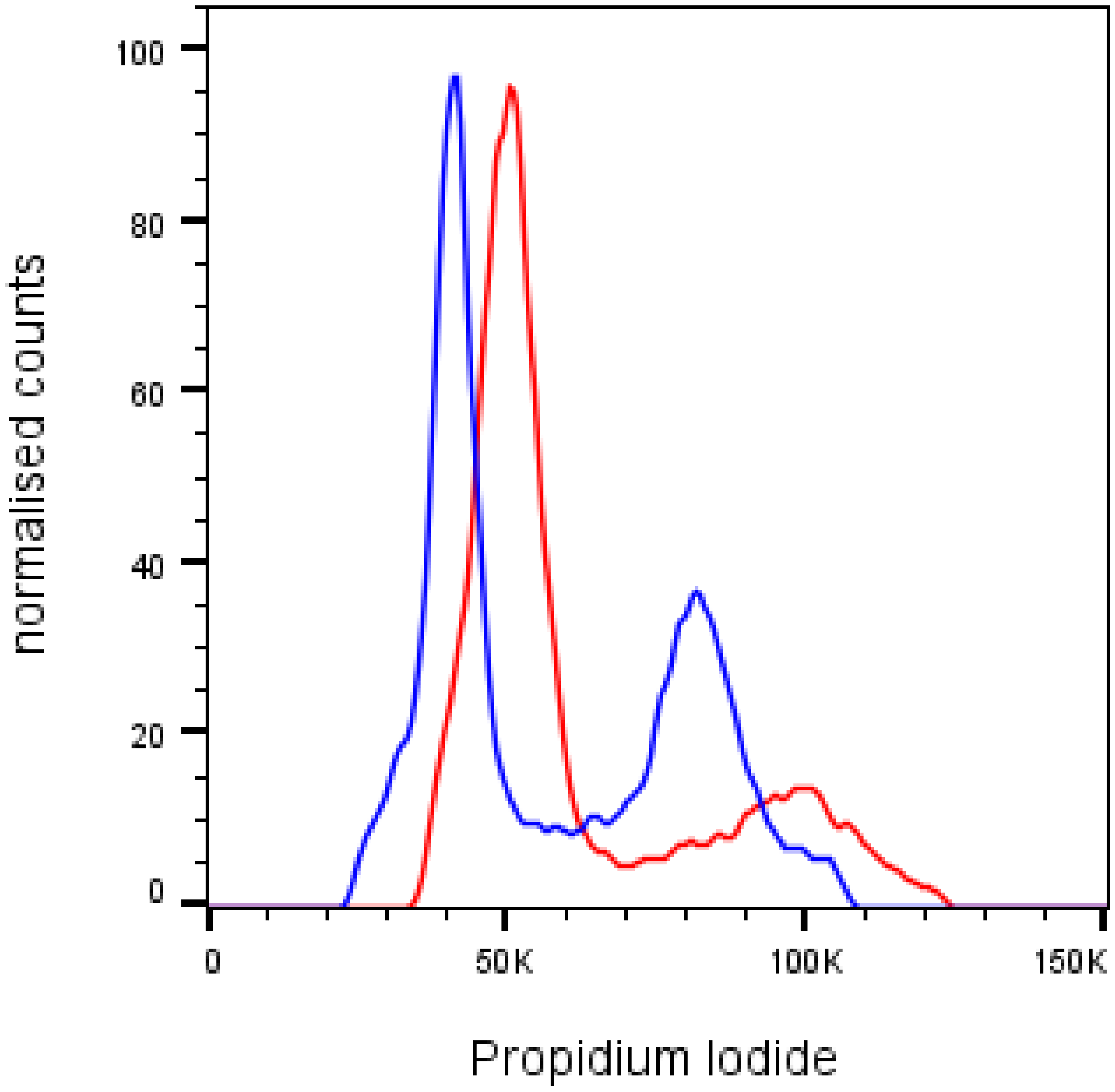

Figure S1: Genome size analysis by flow cytometry of propidium iodide-stained *Trichomonas vaginalis* IR 78 and *T. foetus* KV-1. Overlay of fluorescence intensity plots of propidium iodide stained cells. Blue: *T. foetus* KV-1, red: *T. vaginalis* IR 78.
